# A randomized controlled trial of mental health interventions for survivors of systematic violence in Kurdistan, Northern Iraq

**DOI:** 10.1186/s12888-014-0360-2

**Published:** 2014-12-31

**Authors:** Paul Bolton, Judith K Bass, Goran Abdulla Sabir Zangana, Talar Kamal, Sarah McIvor Murray, Debra Kaysen, Carl W Lejuez, Kristen Lindgren, Sherry Pagoto, Laura K Murray, Stephanie Skavenski Van Wyk, Ahmed Mohammed Amin Ahmed, Nazar M Mohammad Amin, Michael Rosenblum

**Affiliations:** Center for Refugee and Disaster Response and Department of International Health, Johns Hopkins Bloomberg School of Public Health, 615 N. Wolfe Street, Room E8646, Baltimore, MD 21205 USA; Department of Mental Health, Johns Hopkins Bloomberg School of Public Health, 624 N. Broadway, 8th Floor, Baltimore, MD 21205 USA; Heartland Alliance International, 208 S. LaSalle Street, Suite 1300, Chicago, IL 60604 USA; Fine Arts Institute, University of Sulaimani, Kurdistan Region, Iraq; Department of Psychiatry & Behavioral Sciences, University of Washington, 1100 NE 45th Street, Suite 300, Seattle, WA 98105 USA; Department of Psychology, Center for Addictions, Personality, and Emotion Research (CAPER), University of Maryland, College Park, MD 20742-5141 USA; Center for the Study of Health and Risk Behaviors, University of Washington, 1100 NE 45th, Suite 300, Seattle, WA 98105 USA; Division of Preventive and Behavior Medicine, Department of Medicine, University of Massachusetts Medical School, 55 Lake Avenue North, Worcester, MA 01655 USA; Trauma Rehabilitation and Training Center, and Department of Community Health, Sulaimania Polytechnic University, Sulaimania, Kurdistan Region, Iraq; University of Sulaimani, Kurdistan Region, Iraq; Department of Biostatistics, Johns Hopkins Bloomberg School of Public Health, 615 N. Wolfe St., Room E3616, Baltimore, MD 21205 USA; Applied Mental Health Research Group, Center for Refugee and Disaster Response, Johns Hopkins University Bloomberg School of Public Health, c/o 159 Tilden Road, Scituate, MA 02066 USA

**Keywords:** Trauma treatment, Iraq, Task sharing, Evidence-based treatments

## Abstract

**Background:**

Experiencing systematic violence and trauma increases the risk of poor mental health outcomes; few interventions for these types of exposures have been evaluated in low resource contexts. The objective of this randomized controlled trial was to assess the effectiveness of two psychotherapeutic interventions, Behavioral Activation Treatment for Depression (BATD) and Cognitive Processing Therapy (CPT), in reducing depression symptoms using a locally adapted and validated version of the Hopkins Symptom Checklist and dysfunction measured with a locally developed scale. Secondary outcomes included posttraumatic stress, anxiety, and traumatic grief symptoms.

**Methods:**

Twenty community mental health workers, working in rural health clinics, were randomly assigned to training in one of the two interventions. The community mental health workers conducted baseline assessments, enrolled survivors of systematic violence based on severity of depression symptoms, and randomly assigned them to treatment or waitlist-control. Blinded community mental health workers conducted post-intervention assessments on average five months later.

**Results:**

Adult survivors of systematic violence were screened (N = 732) with 281 enrolled in the trial; 215 randomized to an intervention (114 to BATD; 101 to CPT) and 66 to waitlist-control (33 to BATD; 33 to CPT). Nearly 70% (n = 149) of the intervention participants completed treatment and post-intervention assessments; 53 (80%) waitlist-controls completed post-intervention assessments. Estimated effect sizes for depression and dysfunction were 0.60 and 0.55 respectively, comparing BATD participants to all controls and 0.84 and 0.79 respectively, compared to BATD controls only. Estimated effect sizes for depression and dysfunction were 0.70 and 0.90 respectively comparing CPT participants to all controls and 0.44 and 0.63 respectively compared to CPT controls only. Using a permutation-based hypothesis test that is robust to the model assumptions implicit in regression models, BATD had significant effects on depression (p = .003) and dysfunction (p = .007), while CPT had a significant effect on dysfunction only (p = .004).

**Conclusions:**

Both interventions showed moderate to strong effects on most outcomes. This study demonstrates effectiveness of these interventions in low resource environments by mental health workers with limited prior experience.

**Trial Registration:**

ClinicalTrials.Gov NCT00925262. Registered June 3, 2009.

**Electronic supplementary material:**

The online version of this article (doi:10.1186/s12888-014-0360-2) contains supplementary material, which is available to authorized users.

## Background

Survivors of systematic violence, including torture, are at risk of multiple poor mental and physical health outcomes. A meta-analysis of 181 studies of mental health outcomes in conflict-affected or displaced populations found that report of torture increased the odds of PTSD and depression [1]. This meta-analysis and a study of Norwegian immigrants from multiple countries found physical torture the most robust predictor of mental distress [1,2]. Iraqi refugees in the US who reported torture had 4.32 and 2.08 times the odds of mental distress and physical health problems respectively compared to those who had not experienced torture [3]. Though less prevalent than PTSD or depression, generalized anxiety is also frequently reported among survivors of torture [4-7].

A recent review identified 40 treatment studies for survivors of torture and other systematic violence including 11 randomized controlled trials (RCTs) [8]. Only five RCTs (and three quasi-experimental studies) were from low or middle income countries and only one (quasi-experimental study) was in the Middle East. Most treatments were delivered outside clients’ country of origin, focused on posttraumatic stress, and consisted of Cognitive Behavioral Therapy (CBT), Narrative Exposure Therapy (NET), multidisciplinary rehabilitation, or outpatient psychiatry. Effect sizes ranged from 0.51-3.46 for PTSD and 0.56-2.30 for depression [8].

Systematic violence, both physical and psychological, is frequent in Iraqi Kurdistan’s recent history. Saddam Hussein’s government conducted a campaign of persecution and genocide (‘the Anafal’) against Iraqi Kurdistan including bombing, chemical warfare, and forced dislocations [9,10]. Between 1986-9, 50,000-100,000 persons were killed and 4,000 villages destroyed [11-13]. A chemical attack on Halabja city killed 5,000 civilians [9,11,13]. Arbitrary imprisonment, torture, and mass killings were common [14,15].

Kurdistan is now an autonomous region within Iraq, composed of three governorates: Dohuk, Erbil, and Sulaimaniyah. At the time of writing, the region is once again in conflict with substantial nearby areas under control of the Islamic State of Iraq and the Levant (ISIL). At the time of the study Kurdistan was experiencing relatively little violence. However, a qualitative study conducted during the year prior to trial commencement found that those who lived through the 1980s suffer a range of problems that they attribute to the trauma experienced during that time, to their current situation, and to their perception of how they are treated by others. Many respondents complained of being treated poorly by the community and even family; that the suffering and efforts of those who had experienced violence under the Saddam regime were not respected. Their current problems included poverty, discrimination, stigma, mental distress, and reduced social, physical, and economic function [16].

The study’s primary objective was to assess the acceptability, feasibility, and effectiveness of scalable mental health treatments for survivors of systematic violence. We conducted a randomized controlled trial (RCT) in the governorate of Erbil and Sulaimaniyah (including the city of Baquba) comparing Behavioral Activation Treatment for Depression (BATD) and Cognitive Processing Therapy (CPT) to a waitlist-control condition among survivors of systematic violence. Interventions were provided by community mental health workers (CMHWs) in government primary health care clinics. The primary outcome measures were depression and functional impairment. The decision to focus on depression as the primary mental health outcome was based on a prior brief qualitative study identifying current problems of survivors of systematic violence. That study suggested that among survivors’ the most salient current psychosocial problems were related to depression while other problems, such as PTSD symptoms, anxiety and traumatic grief, were less so [16].

## Methods

### Trial design

The original study design included a single trial of three interventions - two evidence-based treatments: Behavioral Activation Treatment for Depression (BATD) and Cognitive Processing Therapy (CPT), and a third treatment condition consisting of a basic supportive counseling program. The trial was to extend across all three governorates of Suleimaniyah, Erbil, and Dohuk, based on a misconception that the populations and situations of the governorates were sufficiently similar. However, we subsequently realized that there are important differences between the populations in Dohuk governorate and the rest of Kurdistan. These differences include language, with the Dohuk governorate speaking a different Kurdish dialect than the other parts of Kurdistan, degree of religiosity, with Dohuk being more religious and politically conservative than the other parts of Kurdistan, and the types of trauma exposures given the proximity of Dohuk to the Turkish border. We therefore changed the design to two separate trials: a trial of CPT and BATD in Erbil and Sulaimaniyah governorates only and a separate trial of basic supportive counseling in Dohuk governorate only (the latter is not reported here).

The two evidence-based treatments, BATD and CPT, were implemented in clinics throughout Erbil and Sulaimaniyah governorates. Our original study design had each intervention providing treatment and control participants at a 3:1 ratio, which would have allowed us to pool all controls (including those from the Dohuk region) resulting in a 1:1 allocation of treatment to controls for each treatment condition. With the decision to evaluate the basic supportive counseling program in the Dohuk region as a separate trial (not reported here), the clinics in Erbil and Sulaimaniyah formed the three-arm RCT reported here: BATD, CPT, and waitlist control. This change was made prior to data collection but after design and budgeting.

Intervention training and supervision was based on the Apprenticeship Model described elsewhere [17]. Briefly, US-based trainers provided a two-week training in BATD or CPT to CMHWs and local supervisors. Supervisors then gave ongoing training and supervision to CMHWs while receiving weekly training and oversight from US-based trainers by phone, Skype, and email. Interventions were provided on a rolling basis between June 2009 and August 2010. The final post-intervention assessment was conducted in January 2011.

Based on a prior qualitative study with Kurdish torture survivors [16], we designated depression symptoms and dysfunction as the primary study outcomes. Other problems identified in that study formed secondary outcomes: posttraumatic stress, anxiety, and traumatic grief.

#### Instrument development and testing

Qualitative study data were used to adapt the Hopkins Symptom Checklist for Depression and Anxiety (HSCL-25) [18,19], the Harvard Trauma Questionnaire (HTQ) [20], and the Inventory of Traumatic Grief [21,22] to measure symptoms of depression, anxiety, posttraumatic stress and traumatic grief. Adaptation included adding 13 locally relevant symptoms. Table 1 presents all the mental health symptoms included in the final instrument, identifying which are from the standard measures and which came from the qualitative study. Participants reported symptom frequency for the prior two-weeks using an ordinal scale of 0 (never) to 3 (always). Analyses of intervention impact on mental health outcomes used mean item scores for each scale, therefore also ranging from 0-3.

**Table 1:** **Mental health scales defined**

**Depression**	**Posttraumatic stress**		**Anxiety**	**Traumatic grief**
- Low in energy, slowed down	- Hopeless about the future	- Others are hostile to you*	- Suddenly scared for no reason	- Hearing voice of deceased person speaking to you
- Blaming self for things	- Loss of interest in things	- Feeling you have no one to rely on*	- Fearful	- Seeing deceased person standing in front of you
- Crying easily	- Trouble sleeping	- Finding out you have done something you cannot remember*	- Faintness	- Feeling you have lost your sense of control
- Loss of sexual interest or pleasure	- Recurrent thoughts or memories of events	- Feeling split into two people, one is watching what the other is doing*	- Nervousness	- Feeling the death of someone close has changed your world view
- Poor appetite	- Feeling events happening again	- Feeling betrayed*	- Heart pounding or racing	- Having pain same part of your body or same symptoms as people who have died
- Difficulty sleeping	- Nightmares	- Unable to express feelings*	- Trembling	- Feeling moving on with your life would be difficult
- Hopeless about the future	- Unable to feel emotions	- Fighting with others*	- Feeling tense	- Envious of others who have not lost someone
- Depressed	- Jumpy, easily startled	- Blaming self for things*	- Headaches	- Lost ability to care about others
- Lonely	- Difficulty concentrating	- Tense*	- Episodes of terror or panic	- Drawn to places and things associated with people who have died
- Thoughts of ending your life	- Avoiding activities that remind of events		- Feeling restless, can’t sit still	- Imitating behaviors of people who have died
- Feeling not free or caught	- Inability to remember parts of events			- Feeling as if already dead
- Worrying to much about things	- Avoiding thoughts/feelings associated with events			- Waiting for dead relatives to come back*
- Loss of interest in things	- Suddenly feeling very different emotionally or physically when reminded of events			
- Everything you do is difficult	- Irritable or outbursts of anger			
- Inferior to others	- Lost ability to care about other people			
- Feeling desperate*	- Feeling people do not understand what happened to you*			
- Wishing you were dead*	- Difficulty performing work or daily tasks*			
- The brain is tired*	- Guilty for having survived*			
- Unable to enjoy feasts or other celebrations*	- Ashamed that events happened to you*			
- Thinking too much*	- Spending time thinking why events happened to you*			
	- Feeling as if going crazy*			
	- Feeling you are the only person who has suffered these events*			

*Indicates items that were added to the standard measures for each syndrome based on a qualitative study (16).

The study instrument also included locally developed function scales for men and women based on frequently mentioned activities in the qualitative study that men/women do to care for self, family, and community. Respondents rated difficulty on an ordinal scale of 0 (‘no more difficulty than most men/women of the same age’) to 4 (‘frequently unable to do the activity’). Analyses of intervention impact on dysfunction used mean item scores for this scale, therefore also ranging from 0-4. Table 2 presents the items included in the function scales for men and women.

**Table 2:** **Function scales defined**

**Function items**	**Function items**
**Men and women**	**Sex specific**
- Exchanging ideas with others	*Males*:
- Having harmonious relationship with wife/husband and family	- Providing for the family
- Bringing up children correctly	- Looking after family behaviors
- Doing things to improve the community	- Labor
- Sympathizing with others	- Giving advice to family members
- Visiting and socializing with others in community	- Giving advice to other community members
- Asking for or getting help when you need it	*Females*:
- Making decisions	- Housework
- Taking part in family activities	- Cooking
- Taking part in community activities	- Other types of manual labor
- Learning new skills	- Caring for family members
- Concentrating on your tasks and responsibilities	- Giving advice to others
- Interacting with people you do not know	
- Attending mosque or religious gathering	
- Assisting others	

Instrument reliability and validity were tested for all outcomes among local survivors of systematic violence (N = 128). Interviewees were taken from lists of torture survivors provided by a local former prisoners association. Persons on these lists had previously been contacted by the interviewers and asked whether they would agree to be interviewed. For the purpose of testing they were asked whether they felt they were depressed, anxious, or experienced excessive fear (the latter term describing symptom similar to posttraumatic stress). Another adult in the house was asked the same questions about the survivor. Those survivors for whom they and the other adult agreed as to the presence or absence of these problems were selected from the lists for interview. This study was done in the year prior to the trial commencement and since the study criteria were the same some of the participants were also subsequently trial participants. Cronbach’s alphas ranged from .725 to .928, indicating adequate internal reliability. Pearson correlation coefficients for test-retest reliability (repeat interviews were conducted 1-3 days later) ranged between .728 and .864. Criterion validity was assessed using methods described elsewhere [23]. Briefly, criterion validity was assessed by comparing the previous statements of the survivors and others as to whether the survivors had depression, anxiety or excessive fear with the results of the instrument measuring the similar concepts of depression, anxiety, posttraumatic stress, and traumatic grief. A high level of agreement would be expected if people can accurately recognize the presence of these problems and if the instrument has criterion validity. Criterion validity was supported for all scales among men but only for posttraumatic stress symptoms among women. However, based on the results on the other tests of validity and reliability measures by sex we decided to retain the measures. Another reason for retention was that we suspected problems with the criterion validity testing procedure. In previous studies we have found that instruments tend to perform either well or poorly across most tests, so poor criterion validity in the face of adequate performance on other measures was unusual. Our local partners speculated that criterion validity was poor among women because their husbands may not be good judges of their wife’s mental state. Detailed descriptions of instrument development are provided elsewhere [24].

#### Intervention selection

The authors consulted torture, trauma, and cross-cultural mental health experts and local stakeholders and mental health providers. Experts disagreed on whether an intervention could address depression without addressing trauma. The PI (PB) decided to evaluate both types of interventions by selecting BATD (depression only treatment) and CPT (trauma and depression).

### Interventions

Two evidence-based treatments were included in this study. The process to adapt both interventions to the local context was iterative, continuing throughout training and early implementation, with feedback sought and incorporated from multiple sources. Both interventions were adapted for low literacy and administration by paraprofessionals. This was the first time BATD or CPT had been adapted in this way.

#### Brief behavioral activation treatment for depression (BATD)

BATD is an empirically supported psychotherapy for depression. It is published in a 12 session format [25] which is briefer than other variants of behavioral activation which often exceed 20 sessions [26] and focuses on strategies to engender structured engagement in healthy and positive values-based behaviors [27-29]. BATD has demonstrated efficacy in eight RCTs in diverse populations [30-33]. Preliminary evidence suggests that the full version of BA may also reduce PTSD symptoms [34-37].

The core content of BATD is based on helping individuals plan for and engage in positive activities on a daily basis based on the values and goals of that individual in multiple life areas (e.g., relationships, career, and spirituality). Engagement in these activities is initially supported by the structure of the program and reinforced by the therapist, but the reinforcement becomes more intrinsic as the activities lead to more positive life experiences and the satisfaction that comes with living according to one’s own values and goals. The treatment was highly consistent with the original treatment manual but with two types of adaptations: First, changes based on cultural issues including a) consideration of societal expectations regarding acceptable and unacceptable activities and b) modification of the discussion of values to be less individual-focused and more collective, focused on the participant’s place in the larger society. Second, changes to address patient adversities including limited language/writing proficiency and extreme poverty. These included a set of stickers developed for patients to use in place of written monitoring of activities, and focus on identifying low cost easily accessible activities to reduce financial barriers.

#### Cognitive processing therapy (CPT)

CPT is a12-session psychotherapy that includes cognitive restructuring (i.e., identifying, challenging, and modifying maladaptive beliefs) and emotional processing of traumatic events (i.e., using a writing narrative to help clients identify and feel their emotions about their worst traumatic events). Cognitive restructuring initially focuses on rigid or inaccurate beliefs about the trauma itself (i.e., reasons why it happened or what could have prevented). It then shifts to overgeneralized beliefs about the self or other that have been affected by the trauma in the domains of safety, trust, power/control, esteem, and intimacy. The ultimate goals of treatment are for clients to be able to approach (vs. avoid) their feelings about the trauma and to modify rigid, inaccurate, or overgeneralized trauma-related beliefs to be more flexible, accurate, and adaptive. Clients are taught these skills throughout the course of treatment and practice outside of session (i.e, homework) is a therapy expectation. CPT was designed to reduce PTSD symptoms in sexual violence survivors [38]. Multiple studies have examined CPT’s effectiveness among female rape victims [39-43]; child sexual abuse survivors [44]; combat veterans [45,46]; physical assault survivors [43]; and refugees [47]. CPT has proven effective in reducing PTSD and depression symptoms compared to controls [41,42,45,46] and reducing PTSD compared to minimal attention [40,44]. Improvements in PTSD and depression symptoms among rape survivors are comparable to Prolonged Exposure Therapy [40]. Unlike the current study previous studies used PTSD diagnosis as an inclusion criterion.

A full description of the adaption of CPT to the Kurdish population can be found elsewhere [48]. Briefly, while the essential elements of CPT were not changed, we adapted training materials to the local culture and situation. Throughout the training manual, reference to American culture-specific examples or explanations were changed to examples relevant to Kurdistan. The final sessions of CPT focus on themes considered related to the experienced trauma: *safety*, *trust*, *power*, *esteem* and *intimacy*. Through discussions with the local CMHWs and mental health professional staff it became clear that no direct translations for *esteem* or *intimacy* were available in the Kurdish language so the alternative themes of *respect* and *caring* were identified that were more culturally appropriate and relevant to the study population. We also adapted the homework assignments to account for the limited literacy levels of the study population. We reduced the complexity and length of all written material and included images as visual cues for the skills being to taught (e.g., reducing the number of questions to challenge maladaptive beliefs from 10 to 5, using images of emotions to cue clients to identify their feelings in relation to a thought). For clients who were illiterate, we also made use of skills some CMHWs had previously learned from our partner, Heartland Alliance. These included rote memorization drills on the skill/assignment for clients to practice over the next week, using mobile phones to record homework, and involving family members as scribes (if there was a family member that the client trusted. No other content specific changes were made to the standard CPT protocol.

#### Waitlist control condition

Study participants randomized to the control condition were informed that they would be waitlisted for treatment and then offered the treatment after approximately 5 months regardless of their re-interview results. During the wait period, CMHWs contacted controls monthly to enquire generally about the severity of their symptoms, particularly that they were not a danger to self or others, as a safety check. Controls were instructed to contact the CMHWs if symptoms substantially worsened, for assessment for possible transfer and referral to a psychiatrist or a torture treatment center in Sulaimaniyah.

### Study settings

The study was implemented in two governorates of Iraqi Kurdistan; Erbil and Sulaimaniyah. Both treatment conditions, BATD and CPT, were implemented in both governorates. Assessments, obtaining informed (verbal) consent, and treatment were performed by CMHWs at 14 Ministry of Health primary health clinics and one outpatient clinic, all in rural areas of the two governorates. CMHWs were nurses, pharmacist assistants, or physician assistants employed by the clinics. All had completed high school and had varying amounts of post graduate and/or job specific training. Criteria for selection were that they worked in government clinics in rural areas, that they had previously been trained by our partner - Heartland Alliance International in basic supportive counseling, and that they were seeing clients before the study. The CMHWs in the study were 7 women and 13 men, aged between 30 and 50 years. Each clinic provided private space for screening and treatment.

### Study eligibility screening and randomization

Recruitment occurred from May 2009 to June 2010. Participants were identified through referral by doctors and nurses at the participating Ministry of Health primary care clinics and through collaboration with former prisoner organizations who notified their members that the services were available. Eligible persons were survivors of systematic violence living in the governorates of Erbil or Sulaimaniyah, aged 18 or over, fluent in Sorani Kurdish, reported significant depression symptoms on the adapted HSCL-25, had no current psychotic symptoms or active suicidality, and appeared mentally competent to consent. ‘Survivor of systematic violence’ was defined as experiencing and/or witnessing physical torture, imprisonment (where torture and other abuse were frequent), and/or military attacks. The latter included gas attacks in the city of Halabja. Many participants were illiterate; half reported no education.

Inclusion in the trial was based on symptom presentation using an adaptation of the HSCL-25 that included the 15 standard depression items plus five local depression-like symptoms identified during the qualitative study. Respondents reported how frequently they experienced each symptom in the prior 2 weeks ranging from 0 to 3. To identify participants with sufficient depression severity warranting treatment, the inclusion criteria included a score of 2 or 3 (equivalent to experiencing a symptoms often or always) on at least one of the DSM-IV A Criteria related to presence of depressive symptoms or anhedonia (crying or feeling depressed most of the time, loss of interest in sex or things generally, or inability to enjoy festivals and celebrations) and a total symptoms score of at least 20. The choice of 20 was arbitrary as we had no prior data on this population. It was chosen because the depression instrument had 20 questions. Therefore a score of 20 or more would indicate symptomatology on a significant proportion of the instrument (at least 7 symptoms). If eligible, the CMHW obtained informed verbal consent and explained that participants would be randomized to immediate treatment (BATD or CPT) or waitlist. If a person consented the CMHW opened a sealed envelope attached to the consent form containing the participant’s assignment. Exclusion criteria were inability to be interviewed due to a cognitive or physical disability, or severe suicidal ideation or behavior.

We used a two-tier randomization process. First 20 CMHWs who worked at primary clinics throughout rural Erbil and Sulaimaniyah governorates were randomized to receive training in either BATD (n = 11) or CPT (n = 9). This resulted in both treatments (BATD and CPT) being equally distributed across both governorates. The second-tier randomization happened at the level of the study participant. Study participants were randomized to study condition (treatment or wait-list control) by the CMHW they saw at their local primary care center where they went for treatment. The CMHWs received twenty participant IDs randomly assigned to intervention or control in the ratio of 3:1 of treatment to wait-controls. Randomization of CMHWs and participant IDs was done by JB using Stata’s randomization function. Investigators kept a master list of each study ID’s assignment for checking randomization fidelity.

### Post-intervention assessment

The study design called for the study instrument to be re-administered by supervisors and CMHWs approximately three to five months after recruitment, following treatment completion. Due to logistical challenges resulting in clients taking longer than expected to complete the treatment (i.e. re-scheduling of sessions due to work or travel), the mean follow up time was 5.5 months, with a range of 1.6-15.5 months. CMHWs or supervisors blind to participants’ treatment status did 197 (85%) of the interviews; 35 (15%) were implemented by un-blinded CMHWs. The latter group included participants who terminated treatment and refused further contact. Rather than forgo assessment, the treating CMHW did the interview.

### Sample size

Sample size was based on a 20% greater reduction in mean depression score in each intervention compared with all controls, power = 0.80, and alpha = 0.05. This yielded 85 participants in each arm, increased to 106 anticipating a 25% drop-out rate. The sample size for each treatment condition was met, but the control sample was smaller than originally planned due to the removal of the participants from the Dohuk region. A post-hoc power analysis using a simplified multiple regression model identified power greater than 0.80 for the primary outcomes of depression and dysfunction comparing CPT to all controls and BATD to all controls. A similar analysis to examine power to detect differences when comparing each treatment to their own controls identified insufficient power (0.40) only for CPT compared to CPT controls for the depression outcome.

### Analysis

All analyses were conducted on the full intent to treat sample and based on change in mean scale scores between baseline and post-assessment. Primary outcomes were mean depression and dysfunction scores. Secondary outcomes were mean anxiety, post-traumatic stress, and traumatic grief scores.

We first compared BATD and CPT to all controls, per the original study design. This relies on the homogeneity assumption that each patient’s outcome is a random draw from a common distribution independent of site. We had no reason to believe that this assumption would be violated because we randomly allocated CMHWs to interventions and recruitment and randomization were consistent across sites. However, a post-hoc analysis of participant characteristics suggested site-specific differences between treatment arms at baseline, challenging the homogeneity assumption. We therefore did a second analysis comparing BATD only to controls generated by the BATD CMHWs (BATD-controls) and CPT only to controls at generated by CPT CMHWs (CPT-controls). This is less precise due to a smaller sample size but more robust by not making the homogeneity assumption. By way of full disclosure we report both analyses.

To estimate treatment effects we used maximum likelihood mixed-effect regression models with a robust variance estimator. All analyses controlled for participant sex, age, marital status, and disability. Additional variables that differed between treatment and control at baseline or that predicted change in outcome were included as covariates (p < 0.10). Multiple imputation by chained equations accounted for missing scale items and follow up scores among those lost to follow up [49]. Clustering at the levels of CMHW and governorate were reviewed.

Effect sizes reflecting regression adjustments were calculated using Cohen’s d [50], which represents the mean differences across the arms standardized by the baseline pooled standard deviation. Effect sizes are equivalent to a Z-score of a standard Normal distribution (i.e. effect size of 1.0 would mean the mean therapy participant’s symptom score is 1.0 standard deviation above the mean waitlist participant’s symptom score). Regression diagnostics included calculating and assessing the normality of standardized residuals at the CMHW and individual level, as well as for the whole model.

A sensitivity analysis was done to assess whether there was any bias in the results due to inclusion of participants assessed by staff unblinded to treatment condition. For this analysis, we analyzed the primary outcomes of depression and dysfunction without the approximately 15% of the sample that was assessed at follow up by study staff familiar with their treatment allocation.

We conducted a third analysis to independently test the null hypothesis of no effect of treatment by applying Rosenbaum et al.’s permutation-based method, [51] which has been applied to cluster randomized trials of mental health interventions by Small et al. [52]. This general method has the advantage of not needing to rely on regression model assumptions nor the aforementioned homogeneity assumption. We used data from all the participants randomized to BATD or CPT and their respective controls. This last analysis was done to examine whether the study conclusions remain valid even without the homogeneity assumption and the standard assumptions implicit in the regression models of the first two analyses (Additional files 1 and 2).

All analyses were conducted using Stata 12.0 and R [53,54]. The study was approved by Johns Hopkins University’s Internal Review Board and University of Sulaimaniyah College of Medicine’s Ethical Committee.

## Results

### Participation

**Figure 1:**
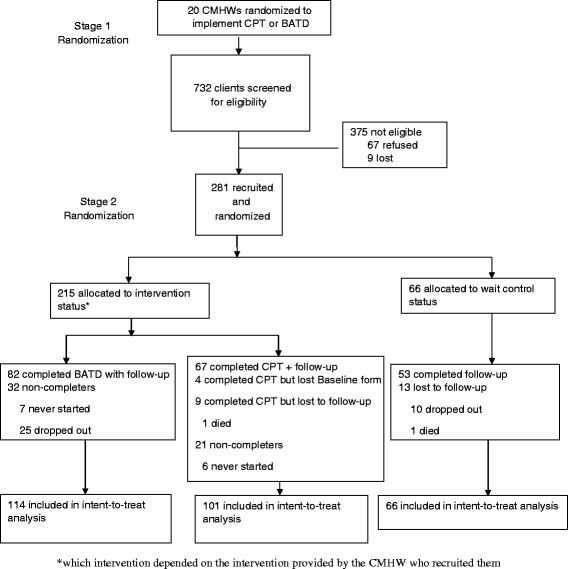
**Flow chart of study participants.**

Of 281 participants, 215 were randomly assigned to intervention (114 to BATD and 101 to CPT) and 66 to waitlist control (Figure 1). Seven BATD participants (6%) never began treatment and 25 (28%) dropped out before completion (nine sessions). Those who did not begin or dropped out of BATD were more likely to be from the Sulaimaniyah governorate, have no education and be self-employed or have irregular work compared with those who completed treatment. Six CPT participants (6%) did not start treatment and 15 (21%) dropped out before completion (also nine sessions). Those who did not begin or dropped out of CPT were more likely to be male and married compared with those who completed treatment. Ten (15%) controls dropped out and 2 (3%) could not be located at follow up. Those controls who were not followed up were more likely to be male, living in Sulaimaniyah governorate, and have at least some education compared with controls who were followed up. Participants who dropped out of the trial after having started the treatment rarely gave reasons beyond not wanting to continue. One CPT and 2 BATD participants left to seek psychiatric help. One CPT participant moved away, one was referred for psychosis, and one left after being verbally abused by her husband for getting treatment. This was the only significant harm or unintended effect reported in the study. One control was referred to a psychiatrist for worsening symptoms.

### Baseline data

A review of demographic characteristics (Table 3) identifies that the proportion of females is smaller among BATD-site controls than the BATD group; the opposite is true for CPT and CPT-site controls. There were also differences in marital status across the groups with the proportion of widows smallest in the BATD group and the proportion of single/divorced the smallest in the CPT control group. Employment status also varied by group, with the CPT controls having the highest proportion of not working participants and the BATD participants having the highest proportion of self-employed or irregular workers.

**Table 3:** **Baseline characteristics**

	**BATD (N = 114)**	**BATD control (N = 33)**	**CPT (N = 101)**	**CPT control (N = 33)**	**All control (N = 66)**
*Demographics*					
Mean age in years	36.9 (12.4)	42.4 (11.1)	41.5 (13.7)	42.3 (14.0)	42.3 (12.5)
Female	65 (57%)	16 (49%)	59 (58%)	23 (70%)	39 (59%)
Location:					
Erbil	50 (44%)	14 (42%)	32 (32%)	11 (33%)	25 (38%)
Sulaimaniyah	64 (56%)	19 (58%)	69 (68%)	22 (67%)	41 (62%)
Marital status:					
Married	76 (67%)	20 (61%)	60 (59%)	21 (64%)	41 (62%)
Single/Divorced^1^	30 (26%)	7 (21%)	24 (24%)	3 (9%)	10 (15%)
Widowed	8 (7%)	6 (18%)	17 (17%)	9 (27%)	15 (23%)
Employment:					
Not working	57 (50%)	17 (52%)	47 (48%)	20 (61%)	37 (56%)
Regular work	25 (22%)	10 (30%)	32 (33%)	10 (30%)	20 (30%)
Self-employed or irregular work^2^	32 (28%)	6 (18%)	18 (19%)	3 (9%)	9 (14%)
Education:					
None	59 (52%)	18 (55%)	44 (44%)	20 (61%)	38 (58%)
Primary	26 (23%)	11 (33%)	30 (30%)	7 (21%)	18 (27%)
Secondary	24 (21%)	4 (12%)	13 (13%)	4 (12%)	8 (12%)
Bachelors/Institutional degree or certificate	5 (4%)	0 (0%)	14 (14%)	2 (6%)	2 (3%)
*Traumatic Experiences*					
Physical torture:					
Experienced personally	43 (38%)	16 (48%)	41 (42%)	16 (48%)	32 (48%)
Witnessed it happen to others	64 (56%)	15 (45%)	45 (46%)	15 (45%)	30 (45%)
Imprisonment:					
Experienced personally	58 (51%)	20 (61%)	62 (64%)	15 (45%)	35 (53%)
Witnessed it happen to others	75 (66%)	20 (61%)	50 (52%)	17 (52%)	37 (56%)
Gas attacks:					
Experienced personally	13 (11%)	4 (12%)	19 (20%)	3 (9%)	7 (11%)
Witnessed it happen to others	16 (14%)	5 (15%)	16 (16%)	4 (12%)	9 (14%)
Other military attacks:					
Experienced personally	71 (62%)	19 (58%)	74 (76%)	23 (70%)	45 (68%)
Witnessed it happen to others	74 (65%)	22 (67%)	61 (63%)	21 (64%)	40 (61%)

^1^Only 3 participants reported being divorced.

^2^Only 12 participants reported being self-employed.

Data on traumatic experiences are missing for 4 people and employment information is missing for 4 people.

Table 4 presents baseline scores for treatment groups and controls. Mean dysfunction and traumatic grief scores are higher among CPT-site participants. Women had higher mean scores across all scales.

**Table 4:** **Baseline mean scale scores by treatment condition** ^**1**^

	**BATD (N = 114)**	**BATD control (N = 33)**	**CPT (N = 101)**	**CPT control (N = 33)**	**All control (N = 66)**
*Primary outcomes*					
Depression					
Total	1.6 (0.5)	1.5 (0.3)	1.7 (0.4)	1.5 (0.4)	1.5 (0.3)
Male	1.4 (0.3)	1.5 (0.3)	1.5 (0.4)	1.3 (0.4)	1.4 (0.3)
Female	1.8 (0.5)	1.7 (0.3)	1.8 (0.4)	1.6 (0.3)	1.6 (0.3)
Dysfunction					
Total	1.7 (0.7)	1.5 (0.5)	2.1 (0.8)	1.9 (0.8)	1.7 (0.7)
Male	1.6 (0.6)	1.4 (0.5)	1.9 (0.8)	1.2 (0.4)	1.3 (0.5)
Female	1.8 (0.7)	1.6 (0.6)	2.2 (0.8)	2.2 (0.8)	2.0 (0.7)
*Secondary outcomes*					
Post-Traumatic Stress					
Total	1.3 (0.5)	1.2 (0.4)	1.4 (0.4)	1.2 (0.3)	1.2 (0.4)
Male	1.1 (0.4)	1.1 (0.4)	1.2 (0.3)	1.0 (0.3)	1.1 (0.4)
Female	1.4 (0.5)	1.3 (0.4)	1.5 (0.4)	1.3 (0.3)	1.3 (0.3)
Anxiety					
Total	1.3 (0.6)	1.2 (0.5)	1.4 (0.5)	1.0 (0.5)	1.1 (0.5)
Male	0.9 (0.4)	1.0 (0.4)	1.2 (0.5)	1.0 (0.5)	1.0 (0.4)
Female	1.5 (0.5)	1.3 (0.6)	1.5 (0.5)	1.1 (0.5)	1.2 (0.6)
Traumatic grief					
Total	0.6 (0.4)	0.5 (0.4)	0.9 (0.4)	0.8 (0.4)	0.6 (0.4)
Male	0.4 (0.3)	0.5 (0.5)	0.8 (0.4)	0.7 (0.5)	0.6 (0.5)
Female	0.8 (0.4)	0.6 (0.4)	1.0 (0.5)	0.8 (0.3)	0.7 (0.4)

^1^Values represent mean (SD) for each scale. For mental health outcomes (Depression, Post-traumatic Stress, Anxiety and Traumatic Grief) the possible scale range is 0-3.0. For the dysfunction scale, the possible scale range is 0-4.0.

### Treatment effects

Treatment effect estimates are presented in Tables 5 and 6. We present CPT and BATD each compared to all controls per our original study design in Table 5. We present CPT to CPT-controls and BATD to BATD-controls in Table 6. Effect sizes for both analyses were medium to large. Depression effect sizes were 0.60 and 0.84 for BATD and 0.70 and 0.44 for CPT compared to all controls and BATD/CPT-specific controls respectively. Depression effect sizes were only statistically significant for CPT compared to all controls and for BATD compared to BATD-site controls. For dysfunction, effect sizes ranged from moderate to large: 0.55 or 0.79 for BATD and 0.90 or 0.63 for CPT based on all controls and BATD/CPT-controls respectively. All were statistically significant. Sensitivity analyses assessing potential bias associated with unblinded follow-up assessments identified small differences in the effect sizes for the primary outcomes but the conclusions were not changed (results not presented). We generated models with and without governorate as a cluster variable and with and without it as a covariate and all 4 models gave very similar results for the outcomes. Therefore the most parsimonious model (the one we used) controlled for clustering within CMHW only.

**Table 5:** **Changes in all study outcomes for CPT and BATD compared with all wait controls** ^**1**^

	**CPT**		**BATD**	
	**Treatment (n = 101)**	**All controls (n = 66)**	**Treatment (n = 114)**	**All controls (n = 66)**
**Primary outcomes**				
*Depression*				
Baseline, mean (se)	1.65 (0.07)	1.60 (0.04)	1.58 (0.07)	1.60 (0.04)
Follow up, mean (se)	0.89 (0.07)	1.16 (0.09)	0.88 (0.10)	1.15 (0.09)
Pre-post change	-0.76 (0.12)	-0.45 (0.10)	-0.71 (0.16)	-0.46 (0.10)
Net effect (95% CI)	-0.31 (-0.54, -0.09)		-0.25 (-0.53, 0.03)	
Effect estimate^2^	0.70**		0.60	
*Dysfunction*				
Baseline, mean (se)	2.02 (0.11)	1.78 (0.14)	1.74 (0.06)	1.71 (0.12)
Follow up, mean (se)	1.14 (0.12)	1.65 (0.12)	1.24 (0.14)	1.59 (0.12)
Pre-post change	-0.88 (0.22)	-0.13 (0.17)	-0.50 (0.17)	-0.12 (0.17)
Net effect (95% CI)	-0.75 (-1.20, -0.30)		-0.38 (-0.71, -0.05)	
Effect estimate^2^	0.90**		0.55*	
**Secondary outcomes**				
*Posttraumatic Stress*				
Baseline, mean (se)	1.32 (0.05)	1.28 (0.05)	1.28 (0.05)	1.28 (0.05)
Follow up, mean (se)	0.72 (0.07)	1.00 (0.07)	0.79 (0.08)	0.99 (0.07)
Pre-post change	-0.60 (0.11)	-0.29 (0.08)	-0.49 (0.13)	-0.29 (0.09)
Net effect (95% CI)	-0.32 (-0.51, -0.12)		-0.21 (-0.43, 0.02)	
Effect Estimate^2^	0.71**		0.47	
*Traumatic Grief*				
Baseline, mean (se)	0.85 (0.03)	0.71 (0.05)	0.67 (0.04)	0.69 (0.06)
Follow up, mean (se)	0.30 (0.07)	0.55 (0.06)	0.41 (0.07)	0.53 (0.06)
Pre-post change	-0.55 (0.08)	-0.16 (0.07)	-0.26 (0.08)	-0.16 (0.07)
Net effect (95% CI)	-0.38 (-0.58, -0.19)		-0.10 (-0.31, 0.10)	
Effect estimate^2^	0.82***		0.24	
*Anxiety*				
Baseline, mean (se)	1.34 (0.06)	1.18 (0.06)	1.25 (0.07)	1.15 (0.05)
Follow up, mean (se)	0.75 (0.10)	0.97 (0.08)	0.75 (0.11)	0.94 (0.08)
Pre-post change	-0.58 (0.11)	-0.21 (0.08)	-0.49 (0.16)	-0.21 (0.09)
Net effect (95% CI)	-0.38 (-0.60, -0.15)		-0.29 (-0.56, -0.01)	
Effect estimate^2^	0.66**		0.53*	

^1^All models included multiple imputation by chained equations for missing data and for missing outcomes due to loss to follow-up. Hierarchical models with robust S.E. estimators were used to account for clustering by CMHW. Covariate selection was done separately by analysis. Additional covariates included were length of time between assessments, any employment vs. no employment, number of children, and baseline dysfunction, depression, anxiety, posttraumatic stress, and traumatic grief score.

^2^Effect sizes measured using Cohen’s *d* statistic calculated using pooled baseline variances.

*p <0.05, **p < 0.01, ***p < 0.001.

**Table 6:** **Changes in all study outcomes for CPT compared with own controls and BATD compared with own controls** ^**1**^

	**CPT**		**BATD**	
	**Treatment (n = 101)**	**CPT-site controls (n = 33)**	**Treatment (n = 114)**	**BATD-site controls (n = 33)**
**Primary outcomes**				
*Depression*				
Baseline, mean (se)	1.64 (0.07)	1.62 (0.06)	1.60 (0.09)	1.60 (0.06)
Follow up, mean (se)	0.92 (0.08)	1.12 (0.15)	0.89 (0.09)	1.25 (0.09)
Pre-post change	-0.72 (0.12)	-0.50 (0.15)	-0.71 (0.16)	-0.35 (0.12)
Net effect (95% CI)	-0.21 (-0.47, 0.04)		-0.35 (-0.50, -0.21)	
Effect Estimate^2^	0.44		0.84***	
*Dysfunction*				
Baseline, mean (se)	2.04 (0.10)	1.98 (0.21)	1.69 (0.07)	1.54 (0.13)
Follow up, mean (se)	1.20 (0.13)	1.70 (0.20)	1.21 (0.13)	1.57 (0.15)
Pre-post change	-0.84 (0.22)	-0.29 (0.25)	-0.48 (0.17)	0.03 (0.22)
Net effect (95% CI)	-0.55 (-1.07, -0.02)		-0.51 (-0.69, -0.33)	
Effect Estimate^2^	0.63*		0.79***	
**Secondary outcomes**				
*Posttraumatic Stress*				
Baseline, mean (se)	1.35 (0.05)	1.33 (0.06)	1.25 (0.09)	1.20 (0.08)
Follow up, mean (se)	0.79 (0.07)	1.05 (0.12)	0.77 (0.07)	0.98 (0.08)
Pre-post change	-0.56 (0.12)	-0.29 (0.13)	-0.48 (0.13)	-0.22 (0.10)
Net effect (95% CI)	-0.27 (-0.48, -0.07)		-0.26 (-0.40, -0.12)	
Effect Estimate^2^	0.61**		0.56***	
*Traumatic Grief*				
Baseline, mean (se)	0.88 (0.03)	0.87 (0.05)	0.62 (0.04)	0.54 (0.08)
Follow up, mean (se)	0.36 (0.06)	0.67 (0.10)	0.35 (0.06)	0.45 (0.06)
Pre-post change	-0.52 (0.08)	-0.21 (0.12)	-0.27 (0.08)	-0.09 (0.05)
Net effect (95% CI)	-0.32 (-0.56, -0.07)		-0.18 (-0.34, -0.02)	
Effect Estimate^2^	0.69*		0.42*	
*Anxiety*				
Baseline, mean (se)	1.33 (0.06)	1.15 (0.05)	1.24 (0.10)	1.23 (0.09)
Follow up, mean (se)	0.77 (0.11)	0.94 (0.12)	0.74 (0.09)	0.99 (0.08)
Pre-post change	-0.56 (0.11)	-0.21 (0.09)	-0.50 (0.17)	-0.24 (0.13)
Net effect (95% CI)	-0.35 (-0.57, -0.12)		-0.26 (-0.48, -0.04)	
Effect Estimate^2^	0.59**		0.48*	

^1^All models included multiple imputation by chained equations for missing data and for missing outcomes due to loss to follow-up. Hierarchical models with robust S.E. estimators were used to account for clustering by CMHW. Covariate selection was done separately by analysis. Additional covariates included in the CPT vs. own controls analysis were length of time between assessments, any employment vs. no employment, any vs. no school, and baseline depression, anxiety, posttraumatic stress, and traumatic grief score. Additional covariates included in the BATD vs. own controls analysis were number of children and any vs. no school.

^2^Effect sizes measured using Cohen’s *d* statistic calculated using pooled baseline variances.

*p <0.05, **p < 0.01, ***p < 0.001.

Effect sizes for secondary outcomes were moderate to large except for BATD compared to all controls (small effect) for traumatic grief and statistically significant except for BATD compared to all controls for posttraumatic stress and traumatic grief.

## Discussion

The third analysis, to independently test the null hypothesis of no treatment effect, identified statistically significant effects for BATD for both primary outcomes, i.e., for depression (p = .003) and dysfunction (p = .007), and for CPT for the outcome of dysfunction only (p = .004).

We studied the impact of BATD and CPT on the primary outcomes of depression and dysfunction and the secondary outcomes of anxiety, posttraumatic stress and traumatic grief symptoms among survivors of systematic violence in Kurdistan. Both interventions were adapted to address local treatment issues including illiteracy and restrictions on women traveling and being treated by male CMHWs. Our original design compared each intervention arm with all controls. However, due to baseline differences between study arms, we also compared BATD to BATD-controls only and did the same for CPT. While estimated effect sizes varied with the type of controls used, both interventions showed moderate to strong impacts on the primary outcomes (depression and dysfunction) in both analyses. The third analysis, which tested the null hypothesis of no treatment effect using a permutation-based method with added robustness to model assumptions, provides additional evidence for BATD for both primary outcomes, and for CPT for the outcome of dysfunction.

Improvement in symptom severity was also evidenced in the waitlist-control group. This is not unusual, particularly given the cyclical nature of mental health symptoms and the expectation that, for some, we may have picked them up at or near the peak of their symptom severity as a consequence of the symptom-level criteria requirement for inclusion in the trial. Similar improvements among waitlist controls have been seen in other controlled trials of psychotherapies in low- and middle-income countries [55,41].

We consider the comparisons to BATD and CPT specific controls (as opposed to the analyses using all the controls combined) as more likely to reflect true intervention impacts. On this basis, effect sizes for CPT on primary outcomes were moderate and moderate to large for secondary outcomes. BATD had a large effect on primary outcomes and moderate effects on all secondary outcomes. Since BATD focuses on behavioral change rather than trauma, this also answered our question of whether a trauma-based approach was necessary to address depression in this trauma-affected population. In terms of the clinical significance of these findings, the study participants started the trial with a mean depression score of about 1.6 indicating that on average, they experienced all the symptoms of depression between a little bit and a moderate amount of the time in the prior 2 weeks. After participating in the intervention, the CPT and BATD participants’ mean scores were all below 1.0, indicating they experienced all the symptoms less than a little bit of the time.

The decision to use depression severity, rather than PTSD symptoms, as the primary mental health outcome was made based on an earlier qualitative study [16] that identified the current priority problems among the target population. RCTs with survivors of systematic violence do not usually focus on depression. One review identified studies that also evaluated and found significant impacts on depression, albeit smaller than PTSD [8]. One study of CBT among ex-detainees (including many torture survivors) found an effect size of 2.0 [56]. Studies of NET for asylum seekers and refugees have produced conflicting results: an effect size of 1.56 in one study [57] and no effect in another [58]. Studies of general counseling and a multidisciplinary intervention have found no effect [59,60]. The effect sizes we found for depression are smaller than some of the trials quoted above [56,57]; however, those trials were all conducted in clinics specifically treating torture survivors and refugees in high-income settings.

RCTs including survivors of systematic violence, including torture, typically focus on PTSD symptoms. Cognitive behavioral therapies [47,56,61,62], NET [57,58] and general psychosocial counseling [63,64] have all demonstrated impact on PTSD symptoms. Effect sizes for cognitive-behavioral and testimony interventions are high, typically 1-3 [47,56-58,61]. There are mixed findings for the effectiveness of general counseling suggesting that it can produce some improvement [63,64]. In our trial, both interventions had a moderate effect on PTSD symptoms.

The generalizability of our conclusions to other populations and contexts is an area for future research. A study by the same researchers and clinical team that used the same methods found much greater effect sizes among survivors of sexual violence in Democratic Republic of Congo [41], suggesting effectiveness may vary by culture and context.

### Limitations

Follow-up occurred within a month following the intervention, preventing evaluation of long-term treatment effects. The study design called for post-assessment three to five months after recruitment, following treatment completion but many clients took much longer than expected to finish treatment so that mean follow up time was 5.5 months. However, while this range may have impacted the results of the trial compared to the original design, the results are therefore probably closer to the results that would be achieved in normal programming. Participants were not blinded to their own treatment/control status. Also, we cannot determine how much of the difference between intervention and control groups is due to regularly meeting with CMHWs regardless of intervention content. 35 out of 232 follow-up interviews were not blinded and therefore subject to possible bias although sensitivity analysis suggests that this was not significant. Instruments showed adequate validity and reliability, however criterion validity was not demonstrated for female respondents, except for PTSD.

Most effect sizes were significant despite a smaller control group than planned. Non-significant effects were moderate in size with upper confidence interval (CI) limits near zero, suggesting that lack of significance was a sample size issue. The exception was BATD compared to all controls for traumatic grief; the effect size was small and the upper CI limit clearly beyond 0, suggesting no appreciable effect.

The number of participants who dropped out from the trial before beginning treatment was relatively small (N = 7 for BATD, N = 6 for CPT). The dropout rates among those who started treatment but did not complete (Figure 1) were 28% and 21%, respectively for BATD and CPT. Drop out rates in CPT trials in high resource countries range from 17-25% (40, 43-46) and 10-25% (30,32,65) in BATD outpatient studies [65]. The similarity between overall non-completion rates in the control and intervention arms (particularly CPT) suggest that the cause is not related to treatment content but to other factors, such as an unwillingness to be contacted regularly.

We lack specific information on the timing and extent of the trauma experiences of the study participants that makes it impossible to link past trauma experiences to current mental health symptoms. Having this information would have allowed a more nuanced investigation of treatment effects, including possible differences by treatment type, by severity of trauma and by time since it occurred.

## Conclusions

This study supports the effectiveness of two psychotherapies for survivors of systematic violence in rural Kurdistan by workers with limited prior experience. Trainers adapted both treatments for illiterate participants. Stigma associated with mental problems was a major issue; families and individuals frequently resisted treatment fearing family reputation and marriageability would be affected. Despite these challenges, the trial demonstrated that CMHWs could implement BATD and CPT. The similarity in drop-out rates between this sample and those in high-resource countries combined with the robust treatment effects suggests that locally adapted CPT and BATD are useful mental health treatments in this setting.

## Additional files

Additional file 1. **CONSORT 2010 checklist of information to include when reporting a randomised trial.**

Additional file 2. **Supplementary Material.**
