# Supplementary material for: A randomized controlled trial of mental health interventions for survivors of systematic violence in Kurdistan, Northern Iraq
Source: BMC Psychiatry. 2014 Dec 31;14:360. doi: 10.1186/s12888-014-0360-2 (PMC4301059; doi:10.1186/s12888-014-0360-2)
Supplement: Supplementary file 1 — CONSORT 2010 checklist of information to include when reporting a randomised trial. [file 12888_2014_360_MOESM1_ESM.docx]

**CONSORT 2010 checklist of information to include when reporting a randomised trial**

| Section/Topic | Item No | | Checklist item | Page No |
| --- | --- | --- | --- | --- |
|  | 1a | | Identification as a randomised trial in the title | 1 |
|  | 1b | | Structured summary of trial design, methods, results, and conclusions | 3-4 |
| Background and objectives | 2a | | Scientific background and explanation of rationale | 5-7 |
|  | 2b | | Specific objectives or hypotheses | 7 |
| Trial design | 3a | | Description of trial design (such as parallel, factorial) including allocation ratio | 7, 14 |
|  | 3b | | Important changes to methods after trial commencement (such as eligibility criteria), with reasons | 7-8 |
| Participants | 4a | | Eligibility criteria for participants | 13 |
|  | 4b | | Settings and locations where the data were collected | 13-14 |
| Interventions | 5 | | The interventions for each group with sufficient details to allow replication, including how and when they were actually administered | 10-12 |
| Outcomes | 6a | | Completely defined pre-specified primary and secondary outcome measures, including how and when they were assessed | 8-9 |
|  | 6b | | Any changes to trial outcomes after the trial commenced, with reasons | N/A |
| Sample size | 7a | | How sample size was determined | 15 |
|  | 7b | | When applicable, explanation of any interim analyses and stopping guidelines | N/A |
| Randomisation: |  | |  |  |
| Sequence generation | 8a | | Method used to generate the random allocation sequence | 14 |
|  | 8b | | Type of randomisation; details of any restriction (such as blocking and block size) | 14 |
| Allocation concealment mechanism | 9 | | Mechanism used to implement the random allocation sequence (such as sequentially numbered containers), describing any steps taken to conceal the sequence until interventions were assigned | 14 |
| Implementation | 10 | | Who generated the random allocation sequence, who enrolled participants, and who assigned participants to interventions | 14 |
| Blinding | 11a | | If done, who was blinded after assignment to interventions (for example, participants, care providers, those assessing outcomes) and how | 14-15 |
|  | 11b | | If relevant, description of the similarity of interventions |  |
| Statistical methods | 12a | | Statistical methods used to compare groups for primary and secondary outcomes | 15-17 |
|  | 12b | | Methods for additional analyses, such as subgroup analyses and adjusted analyses | 16-17 |
| Participant flow (a diagram is strongly recommended) | 13a | | For each group, the numbers of participants who were randomly assigned, received intended treatment, and were analysed for the primary outcome | 18, 44 |
|  | 13b | | For each group, losses and exclusions after randomisation, together with reasons | 18, 44 |
| Recruitment | 14a | | Dates defining the periods of recruitment and follow-up | 8 |
|  | 14b | | Why the trial ended or was stopped | N/A |
| Baseline data | 15 | | A table showing baseline demographic and clinical characteristics for each group | 40-41 |
| Numbers analysed | 16 | | For each group, number of participants (denominator) included in each analysis and whether the analysis was by original assigned groups | 15, 18 |
| Outcomes and estimation | 17a | | For each primary and secondary outcome, results for each group, and the estimated effect size and its precision (such as 95% confidence interval) | 19-20, 42-43 |
|  | 17b | | For binary outcomes, presentation of both absolute and relative effect sizes is recommended | N/A |
| Ancillary analyses | 18 | | Results of any other analyses performed, including subgroup analyses and adjusted analyses, distinguishing pre-specified from exploratory | N/A |
| Harms | 19 | | All important harms or unintended effects in each group (for specific guidance see CONSORT for harms28) | N/A |
| Limitations | 20 | | Trial limitations, addressing sources of potential bias, imprecision, and, if relevant, multiplicity of analyses | 22-24 |
| Generalisability | 21 | | Generalisability (external validity, applicability) of the trial findings | 24 |
| Interpretation | 22 | | Interpretation consistent with results, balancing benefits and harms, and considering other relevant evidence | 24 |
| **Other information** | | | |  |
| Registration | | 23 | Registration number and name of trial registry | 4 |
| Protocol | | 24 | Where the full trial protocol can be accessed, if available | 4 |
| Funding | | 25 | Sources of funding and other support (such as supply of drugs), role of funders | 26 |
