# Supplementary material for: A randomized controlled trial of mental health interventions for survivors of systematic violence in Kurdistan, Northern Iraq
Source: BMC Psychiatry. 2014 Dec 31;14:360. doi: 10.1186/s12888-014-0360-2 (PMC4301059; doi:10.1186/s12888-014-0360-2)
Supplement: Supplementary file 2 — Supplementary Material. [file 12888_2014_360_MOESM2_ESM.docx]

Supplementary Material:

Permutation-Based Tests

We applied the method of Rosenbaum et al. (2002), which has been used to analyze cluster randomized trials of mental health interventions by Small et al. (2008). This general method has the advantage of not needing to rely on regression model assumptions nor the cluster homogeneity assumption. In the framework of Rosenbaum et al. (2002), each participant’s baseline variables and potential outcomes under assignment to each possible arm (BATD, CPT, or control) are considered fixed, while the assignment process of CMHW’s to BATD/CPT and of individuals to treatment/control is random due to the trial design. The outcome for each participant is the change score between baseline and post-assessment (for a given outcome type, such as depression or dysfunction).

For clarity, we first define a test of the null hypothesis of no effect of BATD compared to control, which ignores baseline variables; we then extend this to incorporate information in baseline variables. We focus on all possible assignments of individuals within each BATD CMHW to treatment/control, while holding the other aspects of the randomization procedure fixed, i.e., conditioning on the assignment of CMHWs (clusters) to BATD/CPT, and on the assignment within each CPT CMHW of individuals to CPT/control. By design, within each BATD CMHW, individuals were assigned to BATD/control using a 3:1 randomization ratio; we call this the randomization distribution within each BATD CMHW. Define the test statistic T to be the difference in the sample means comparing those assigned to treatment versus control, pooling all participants who have a BATD CMHW. To compute a p-value for this statistic, we simulated 10000 hypothetical trials from the 3:1 randomization distribution within each BATD CMHW, temporarily reassigning each BATD CMHW participant to treatment versus control, and recomputing the test statistic. The p-value is defined as the proportion of hypothetical trials where the simulated test statistic is greater than the observed value of T.

We extend the above analysis to incorporate baseline variable information, using the approach of Rosenbaum et al. (2002), in order to improve power. We fit a linear regression model for the outcome (change score) given the following baseline variables, which were used as main terms: an intercept, sex, age, marital status, working status, and baseline value of the outcome being considered (e.g., baseline depression). All data were used that had these baseline variables measured, and missing outcomes were adjusted for using a logistic regression model fit for the probability of being missing given baseline variables and treatment assignment. The analysis in the previous paragraph was conducted, except replacing each participant’s outcome variable by the residual under the linear regression model fit. E.g., the test statistic T is now the difference in sample means of residuals comparing those assigned to treatment versus control, pooling all participants with BATD CMHW. The p-values in the paper correspond to this analysis. As shown by Rosenbaum et al. (2002), these p-values remain valid regardless of whether the linear regression model assumption holds or if the clusters are homogenous. An analogous method was used to compare CPT to control.

The above method was done separately within BATD CMHW’s and within CPT CMHW’s, to test the two corresponding null hypotheses. If we had instead focused on testing the joint (also called intersection) null hypothesis that neither BATD nor CPT have any effect compared to control, we could have simultaneously considered reassignments of BATD/CPT to each CMHW, and reassignments of individuals to treatment/control within each CMHW. This would have used outcomes from all treatment and all control participants. However, rejecting this joint null hypothesis would leave unanswered which treatment(s), i.e., BATD, CPT, or both, have a non-zero effect compared to control. For this reason, we tested the two null hypotheses separately, as described above.
